# Carbon-Based Nanomaterials Increase Reactivity of Primary Monocytes towards Various Bacteria and Modulate Their Differentiation into Macrophages

**DOI:** 10.3390/nano11102510

**Published:** 2021-09-27

**Authors:** Tereza Svadlakova, Martina Kolackova, Radka Vankova, Rumeysa Karakale, Andrea Malkova, Pavel Kulich, Frantisek Hubatka, Pavlina Turanek-Knotigova, Irena Kratochvilova, Milan Raska, Jan Krejsek, Jaroslav Turanek

**Affiliations:** 1 Institute of Clinical Immunology and Allergology, University Hospital Hradec Kralove and Faculty of Medicine in Hradec Kralove, Charles University, 50005 Hradec Kralove, Czech Republic; svadlakovat@lfhk.cuni.cz (T.S.); kolackovam@lfhk.cuni.cz (M.K.); vankovr@lfhk.cuni.cz (R.V.); rumeysa.karakale@fnhk.cz (R.K.); 2 Institute of Preventive Medicine, Faculty of Medicine in Hradec Kralove, Charles University, 50003 Hradec Kralove, Czech Republic; Malka8AR@lfhk.cuni.cz; 3 Veterinary Research Institute, 62100 Brno, Czech Republic; kulich@vri.cz; 4 C2P NEXARS, 62500 Brno, Czech Republic; frantisek.hubatka@nexars.com (F.H.); pavlina.turanek@nexars.com (P.T.-K.); 5 Institute of Physics, Czech Academy of Sciences, 18200 Prague, Czech Republic; krat@fzu.cz; 6 Department of Immunology, Faculty of Medicine and Dentistry, Palacky University Olomouc, 77515 Olomouc, Czech Republic; milan.raska@upol.cz

**Keywords:** graphene, carbon nanotubes, cytotoxicity, immunomodulation, inflammation, monocytes, phagocytosis

## Abstract

The evaluation of carbon-based nanomaterials’ (C-BNMs’) interactions with the immune system, notably their ability to cause inflammation, is a critical step in C-BNM health risk assessment. Particular attention should be given to those C-BNMs that do not cause direct cytotoxicity or inflammation on their own. However, the intracellular presence of these non-biodegradable nanomaterials could dysregulate additional cell functions. This is even more crucial in the case of phagocytes, which are the main mediators of defensive inflammation towards pathogens. Hence, our study was focused on multi-walled carbon nanotubes (MWCNTs) and two different types of graphene platelets (GPs) and whether their intracellular presence modulates a proinflammatory response from human primary monocytes towards common pathogens. Firstly, we confirmed that all tested C-BNMs caused neither direct cytotoxicity nor the release of tumour necrosis factor α (TNF-α), interleukin (IL)-6 or IL-10. However, such pre-exposed monocytes showed increased responsiveness to additional bacterial stimuli. In response to several types of bacteria, monocytes pre-treated with GP1 produced a significantly higher quantity of TNF-α, IL-6 and IL-10. Monocytes pre-treated with MWCNTs produced increased levels of IL-10. All the tested C-BNMs enhanced monocyte phagocytosis and accelerated their differentiation towards macrophages. This study confirms the immunomodulatory potential of C-BNMs.

## 1. Introduction

C-BNMs are rightfully considered to be among the most promising tools wielded in the development of modern technology. Their most important properties include electrical conductivity, mechanical strength and a high surface area. As a result, C-BNMs appear to have potential applications in nanomedicine, either as adjuvants or scaffolds, and as carriers of anticancer drugs [1,2,3]. Their ability to penetrate various barriers to enter the human body and bind to various macromolecules has evoked a lot of interest in their medical applications. On the other hand, the same properties make them a potential danger to human health [4,5,6].

An essential step in their toxicity/biocompatibility assessment is an evaluation of their interaction with the immune system. Following their penetration of protective barriers, peripheral immune cells are the first in line to interact with them. C-BNMs, predominantly graphene oxide and carbon nanotubes (CNTs), have been extensively tested for their potential to promote inflammation and cell death [4,6,7,8,9,10]. Recently, other graphene derivates, such as graphene platelets (GPs), have garnered attention [11,12,13,14]. In contrast to CNTs, pristine GPs seem to have lower or no immunopathological effects [11,12,15]. However, it is still necessary to attain advanced immunological data.

Although the most likely sites of exposure to C-BNMs are the pulmonary and gastro-intestinal systems, some studies have pointed out the possibility of their translocation and biodistribution to distant organs, such as the spleen or liver, via the peripheral blood system [16,17,18]. Therefore, research that focuses on the interaction with blood components is necessary to establish a full mosaic of the potential risks. Considering the use of C-BNMs in medical applications, such research is even more essential [3,19,20]. As the nanomaterials may enter the blood system, primary monocytes would be among the first cell types exposed to them. This heterogeneous group of mononuclear phagocytes, along with their differentiated progeny, dendritic cells and macrophages, play both regulatory and effector roles in host defence. Key functions such as phagocytosis and immunomodulation most likely determine the overall impact of the present nanomaterials [21]. Several studies have already pointed to the role of C-BNMs in cytotoxicity [22,23]. However, the immune system works as a highly dynamic system, which in real life balances reactions to more than one stimulus by specific regulations. Thus, it is necessary to consider the possible effect of immunomodulation, especially by those materials that seem to have no acute cytotoxic or even pro/anti-inflammatory effects. Several studies have already suggested this potential immunomodulatory effect, for example, the co-stimulation with iron and polystyrene nanoparticles led to a decrease in the proinflammatory response to lipopolysaccharide (LPS) [24,25]. On the other hand, the co-stimulation of CNTs and pristine graphene with Toll-like receptor (TLR)4 and TLR2 agonists led to the increased release of proinflammatory IL-6 and TNF-α from human mononuclear cells and mouse bone-derived macrophages, respectively [26,27]. In our previous study, we confirmed inflammasome NLRP3’s canonical activation by synergic exposition to GPs and muramyl dipeptide, even though GPs alone did not induce any IL-1β release [11].

Until recently, studies have focused predominantly on the immunomodulative effect of C-BNMs on macrophages of various origins or THP-1 monocytes [27,28,29,30]. However, there is a significantly lower number of studies focused on the modulation of primary human blood monocytes. As mentioned above, circulating monocytes represent a flexible population that is rapidly recruited to sites of infection or tissue injury. Any immunomodulation or even disruption of monocyte function would have a huge impact on inflammation and its regulation at the infection site. Generally, monocytes are extremely sensitive to microbial contamination due to their expression of TLRs, e.g., TLR4 or TLR2, which makes them a suitable cell model for testing immunomodulatory effects. Therefore, we decided to examine whether phagocyted pristine GPs and MWCNTs could modulate subsequent monocyte-mediated phagocytosis and cytokine response towards representative whole bacteria.

## 2. Materials and Methods

### 2.1. Nanomaterials

C-BNMs used in this study included GP1 (PL-P-G750; PlasmaChem GmbH, Berlin, Germany), GP2 (CRANN, Trinity College Dublin, Ireland) and MWCNTs (659258; Sigma-Aldrich, St. Luis, MO, USA). Stock suspensions of both GPs at a concentration of 250 μg/mL and MWCNTs at a concentration of 500 μg/mL were prepared using sonication in 0.02% sodium cholate as previously described [11]. The average shape and size were assessed by transmission electron microscopy (TEM, Philips 208 S Morgagni, FEI, Brno, Czech Republic) at an accelerating voltage of 80 kV and by scanning electron microscopy (SEM, Magellan 400L, FEI, Brno, Czech Republic). All C-BNMs used here have been characterized in detail in our previous study [11]. Their basic characteristics are summarized in Table 1.

#### Biological Contamination

Possible contamination with biologically active TLR4 and TLR2 agonists was assessed with reporter cell assays using HEK-Blue™-4 and HEK-Blue™-2 cells (InvivoGen, San Diego, CA, USA) as they stably express human TLR4 and TLR2, respectively. The stimulation of these receptors leads to the activation of a reporter gene for NF-kB-inducible secreted embryonic alkaline phosphatase (SEAP). Released AP was detected in supernatants using QUANTI-Blue™ (InvivoGen, San Diego, CA, USA), a SEAP detection medium that produces a purple/blue colour.

Both cell lines were maintained in Dulbecco’s modified Eagle’s high glucose medium without phenol red (DMEM; Corning, NY, USA) supplemented with 10% heat inactivated ultra-low endotoxin fetal bovine serum (FBS_LE_; Biosera, France), 2 mM L-alanyl-L-glutamine (GlutaMAX; Life Technologies, Carlsbad, CA, USA), Normocin (100 μg/mL; InvivoGen, San Diego, CA, USA) and selective antibiotics 250X HEK-Blue™ Selection (InvivoGen, San Diego, CA, USA). Cells were seeded in flat-bottom 96-well plates at a density of 5 × 10^4^ cells per well and treated with non-cytotoxic levels of GPs (60 μg/mL) and MWCNTs (30 μg/mL) for 24 h. Ultrapure LPS from *Escherichia coli* K12 (100 ng/mL) and heat-killed *Staphylococcus aureus* (HKSA, 10^7^ cells/mL) were used as controls. The absorbance was measured in a Synergy HTX (Biotek, Bad Friedrichshall, Germany) microplate spectrophotometer at a wavelength of 630 nm. To prevent possible interference with these assays, C-BNMs spiked with LPS (100 ng/mL) or HKSA (10^7^ cells/mL) were tested as well (see Supplementary Materials).

### 2.2. Monocyte Isolation and Culture

Peripheral blood samples were obtained from healthy volunteers after their informed consent and approval by the Ethics Committee, University Hospital Hradec Kralove, Sokolska 581, 500 05 Hradec Kralove (reference number 201902 S22P), Czech Republic. Peripheral monocytes were isolated from whole blood according to the manufacturer’s instructions. Following incubation with 50 μL/mL of RosetteSep™ Monocyte Enrichment Cocktail (STEMCELL Technologies Inc., Vancouver, Canada), gradient density centrifugation (1200× *g*, 20 min, RT) was performed using Histopaque^®^-1077 (Sigma-Aldrich, St. Luis, MO, USA). The purity (~85%, Figure S2) of isolated monocytes was evaluated by flow cytometry (Navios™, Beckman Coulter, Brea, CA, USA). A more detailed description of their isolation and phenotyping is provided in the Supplementary Materials.

### 2.3. Monocyte Exposition to C-BNM

Freshly isolated monocytes were suspended in RPMI 1640 without phenol red (Corning, NY, USA) supplemented with 20% human autologous serum, 2 mM GlutaMAX and Primocin™ (100 μg/mL, InvivoGen, San Diego, CA, USA) and seeded at 2 × 10^6^/mL in a 96-well plate (0.1 mL) or 12-well plate (0.5 mL). Non-attached cells were carefully removed 1 h after seeding. Adhered monocytes were incubated at a final volume of 0.2 mL (96-well plate) or 1 mL (12-well plate) with full RPMI medium (negative control) and either GPs (5–60 μg/mL) or MWCNTs (5–30 μg/mL). To verify the non-cytotoxic effect of the used C-BNMs, monocytes were incubated for 24 h and 48 h. Viability was assessed by LDH assay according to the manufacturer’s protocol. Absorbance was measured on a Synergy HTX microplate spectrophotometer at 490 nm, with 690 nm set as the reference wavelength. Further experiments were performed with 24 h exposure to non-cytotoxic concentrations of GPs (60 μg/mL) or MWCNTs (30 μg/mL).

#### 2.3.1. Oxidative Stress

Following 24 h exposure to C-BNMs, possible oxidative stress of monocytes was evaluated by the glutathione (GSH) concentration of cell lysates using a glutathione colorimetric detection kit (Invitrogen, Thermo Fisher Scientific, Carlsbad, CA, USA). Cells were collected and processed according to the manufacturer’s protocol. Absorbance was measured in a Synergy HTX microplate spectrophotometer at 405 nm. To determine oxidized glutathione (GSSG), lysates were treated with 2-vinilpyridine (2PVP; Sigma-Aldrich, St. Luis, MO, USA) for 1 h at RT. The concentration of total GSH and GSSG was determined according to the standard curves for GSH and GSSG, respectively. The concentration of free GSH was calculated by subtracting the GSSG concentration values from the total GSH.

#### 2.3.2. Intracellular Localization of C-BNM

Monocytes exposed to C-BNMs were collected and fixed in 3% glutaraldehyde. Samples were centrifuged, and the pellet was rinsed in Milonig buffer; post-fixed in 1% OsO_4_ solution in Milonig buffer; dehydrated in 50%, 70%, 90% and 100% ethanol; embedded in Epon–Durcupan mixture (Epon 812 Serva, Heidelberg, Germany; Durcupan, ACM Fluka, Buchs, Switzerland); and polymerized at 60 °C for 72 h. Ultrathin (60 nm) sections were cut with glass knives on UC 7 ultramicrotome (UC 7, Leica, Vienna, Austria) and contrasted with 2% uranyl acetate and 2% lead citrate. The obtained sections were examined using TEM (Philips 208 S Morgagni, FEI, San Jose, CA, USA).

### 2.4. Monocyte Exposition to Bacteria

Following 24 h incubation with C-BNMs, cells were washed with RPMI 1640 to remove unincorporated nanomaterials and were separately treated with heat-killed bacteria *Escherichia coli* serotype 0111:B4 (HKEB), *Staphylococcus aureus* (HKSA) and *Pseudomonas aeruginosa* (HKPA) (InvivoGen, San Diego, CA, USA) at a final concentration of 10^7^ cells/mL for a further 24 h. Cells cultivated only with bacteria without pre-treatment with C-BNMs were used as a control. Viability was determined using LDH assay according to the manufacturer’s protocol.

### 2.5. Cytokine Secretion

#### 2.5.1. IL-6 and IL-10 Production

IL-6 and IL-10 in supernatants of monocytes exposed to C-BNM, bacteria and combined treatments were detected by cell-based assays using human reporter cell line HEK-Blue™ IL-6 cells and HEK-Blue™ IL-10 cells, respectively. Both cell lines were purchased from Invivogen (San Diego, CA, USA). HEK-Blue™ cells were maintained in DMEM supplemented with 10% FBS_LE_, 2 mM GlutaMAX, Normocin and selection antibiotics 250X HEK-Blue™ Selection.

HEK-Blue™ IL-6 cells and HEK-Blue™ IL-10 cell lines are designed to specifically respond to appropriate IL-6/IL-10. The binding of IL to its receptor IL-R on the surface of HEK-Blue™ allows for the specific detection of bioactive cytokines via colorimetric assay based on the enzyme activity of expressed reporter gene SEAP. SEAP was quantified using QUANTI-Blue™, a SEAP detection medium, which turns blue in its presence. Absorbance was measured using a microplate spectrophotometer at 630 nm wavelength.

#### 2.5.2. TNF-α Production

TNF-α in supernatants of monocytes exposed to C-BNM, bacteria and combined treatments was detected using Human TNF-α Quantikine ELISA Kit (R&D Systems, Minneapolis, MN, USA) according to the manufacturer’s protocol.

### 2.6. Evaluation of Phagocytosis

Monocytes exposed to all C-BNMs for 24 h were washed with RPMI 1640 and treated with pHrodo™ Red *E. coli* BioParticles™ (EC; Life Technologies, Carlsbad, CA, USA) at a concentration of 200 μg/mL in cultivation medium. After 3 h of incubation at 37 °C, cells were washed with a phosphate-buffered solution (PBS; Sigma-Aldrich, St. Luis, MO, USA). As the pHrodo™ Red fluorescence increases as pH decreases from neutral to acidic, incorporation of bacteria was observed using a holotomographical microscope Nanolive 3D Cell Explorer-fluo with excitation/emission wavelengths of 560/585 nm with the software STEVE version 1.6.3496 (Nanolive, Ecublens, Switzerland).

Analogously treated cells were washed with PBS containing 1 mM ethylenediamine tetraacetic acid (EDTA), 1% bovine serum albumin (BSA), 2% FBS, and 0.1% sodium azide (NaN_3_) (Sigma-Aldrich, St. Luis, MO, USA). Samples (30,000 events) were then acquired on Flow cytometer Navios™ (Beckman Coulter, Brea, KA, USA) using a 488-nm argon-ion laser and 620/30 FL3 channel. To exclude possible interference of autofluorescence of C-BNMs, cells with C-BNMs but without EC were analysed as well.

### 2.7. Monocyte Differentiation to Macrophages

To investigate whether engulfed C-BNMs influence the differentiation of monocytes, after 24 h incubation with C-BNM, cells were washed and incubated with RPMI 1640 without phenol red supplemented with 10% human autologous serum, 2 mM GlutaMAX and Primocin™ for a further 6 days. On the seventh day, cells were examined under optical microscope Nikone Eclipse Ts2 (Nikon, Osaka, Japan). Detached cells were washed with PBS containing 1 mM EDTA, 1% bovine serum albumin (BSA), 2% FBS and 0.1% sodium azide (NaN_3_) and stained with anti-CD64-PE (Beckman Coulter, Brea, KA, USA) and anti-CD163-FITC (Becton Dickinson, Prague, Czech Republic). The fluorescent signals of samples (60,000 events) were then acquired in 525/40 FL1 and 575/30 FL2 channel by Flow cytometer Navios™ (Beckman Coulter, Brea, KA, USA) equipped with a 488-nm argon-ion laser.

### 2.8. Statistical Analysis

Data are expressed as mean values (*n*-tests = 3) ± standard deviation and are normalized to the control. Unless stated otherwise, based on the Shapiro–Wilk test of normality, either the parametric or nonparametric analysis of variance (ANOVA) followed by Dunnett’s test or Kruskal–Wallis test was performed using GraphPad Prism™ software version 8.2.1 (GraphPad Software Inc., San Diego, CA, USA).

## 3. Results

### 3.1. Nanomaterials

**Figure 1:**
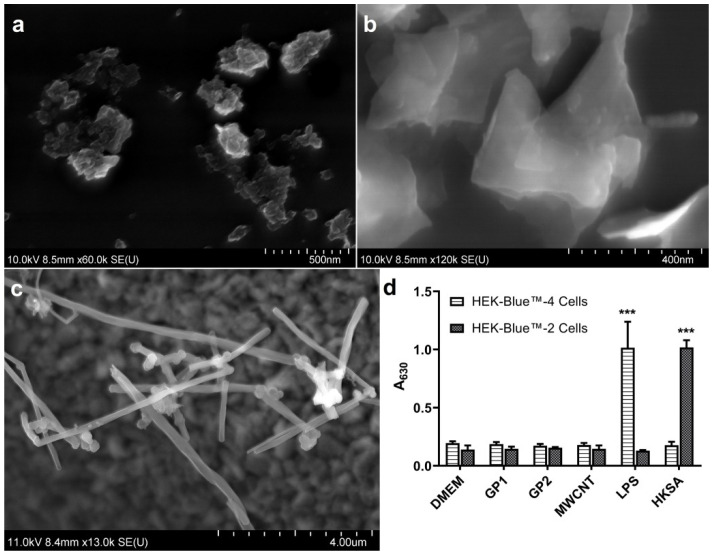
Characterisation of C-BNM: (**a**) SEM image of GP1 forming small aggregates; (**b**) SEM image of GP2 forming large sheets; (**c**) SEM detail of MWCNTs’ structure; (**d**) HEK-Blue™ cells respond to C-BNMs. Data are presented as median with 95% CI. *** *p* < 0.001 highlights statistical significance as compared to untreated control (DMEM).

Detailed characterisations of all used C-BNMs have been provided in our previous study [11]. The basic characteristics of C-BNMs in stock solution are shown in Table 1. Briefly, according to the average diameter and SEM images, both GP1 (Figure 1a) and GP2 (Figure 1b) are heterogeneous in their shape, and they form smaller lump-like flakes and large sheets, respectively. Figure 1c shows that MWCNTs up to 10 μm of length form small clumps.

As shown in Figure 1d, none of the C-BNMs alone activated TLR4 or TLR2 in reporter HEK-Blue™-4 or HEK-Blue™-2 cells, respectively. Both cell lines are highly sensitive (0.01 EU/mL) to the presence of these agonists, which indicates the absence of biological contamination. Moreover, neither GPs nor MWCNTs interfered with the TLR4-NFκB or TLR2-NFκB signal pathways (Figure S1).

### 3.2. Monocyte Exposition to C-BNM

#### 3.2.1. Viability

**Figure 2:**
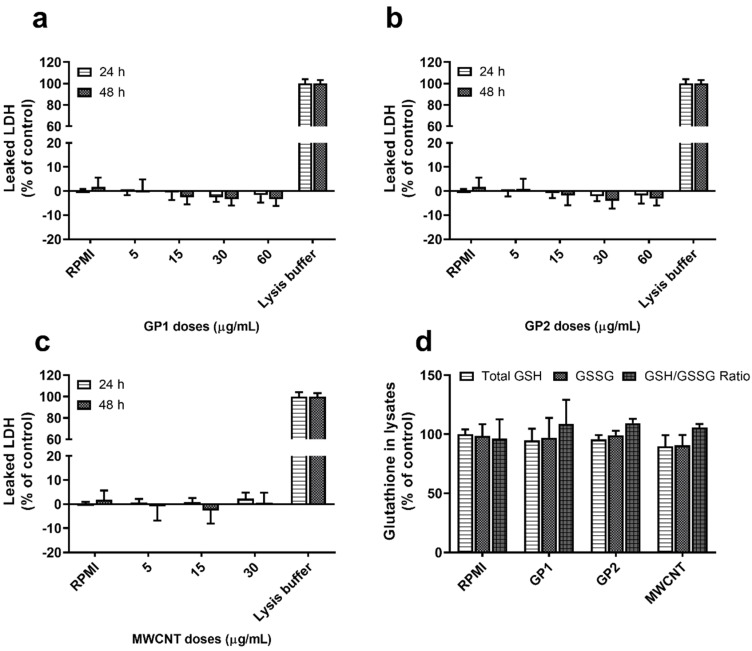
Monocytes’ response to C-BNMs; Viability of monocytes assessed as %-leakage of LDH after 24 and 48 h exposure to (**a**) GP1, (**b**) GP2 and (**c**) MWCNTs. Data are reported as mean ± standard deviation (Leaked LDH % = (T-RPMI)/(L-RPMI) × 100). *T*—test cells, *RPMI*—untreated control, *L*—Lysates. (**d**) Glutathione content in monocyte lysates after 24 h exposure to GPs (60 μg/mL) and MWCNTs (30 μg/mL). Data are presented as % of untreated control (RPMI) and expressed as median with 95% CI.

Viability was evaluated after 24 h and 48 h of monocyte exposure to GP (5–60 μg/mL) and MWCNT (5–30 μg/mL) using the lactate dehydrogenase (LDH) assay. Within these concentration ranges, none of the C-BNMs induced significant cell membrane damage or subsequent release of LDH into the cytoplasm (Figure 2a–c).

The results from the GSH assay have shown that 24 h of incubation with neither GP nor MWCNT led to significant GSH and GSH/GSSG ratio changes (Figure 2d).

#### 3.2.2. Intracellular Localization of C-BNM

**Figure 3:**
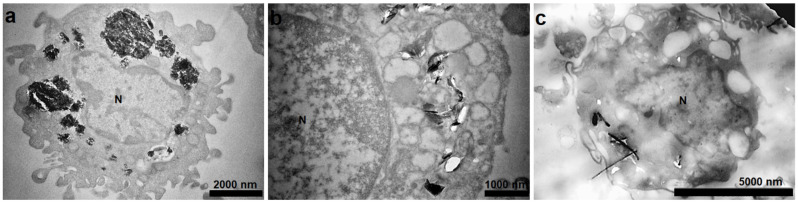
TEM images of intracellular distribution of (**a**) GP1 at 60 μg/mL, (**b**) GP2 at 60 μg/mL and (**c**) MWCNTs at 30 μg/mL after 24 h exposure. N—nucleus.

TEM confirmed the engulfment of C-BNM by monocytes after 24 h of incubation (Figure 3). As can be seen in Figure 3a–c, all C-BNMs were localised in the cytoplasm and no particles were localized in the nucleus. GP1 formed large aggregates enclosed in big endosomes (Figure 3a), whereas GP2 formed smaller aggregates more dispersed within the cytoplasm (Figure 3b). Furthermore, MWCNTs were present mostly as free particles throughout the cytoplasm, where they could possibly interact with multiple organelles (Figure 3c). Individual nanotubes were observed penetrating through the nucleus membrane, although without subsequent obvious cell damage (see Section 3.5. Monocyte Differentiation to Macrophages). Under these conditions, monocytes were subsequently exposed to bacterial treatment.

### 3.3. Monocyte Exposition to Bacteria

#### 3.3.1. Cytokine Production

**Figure 4:**
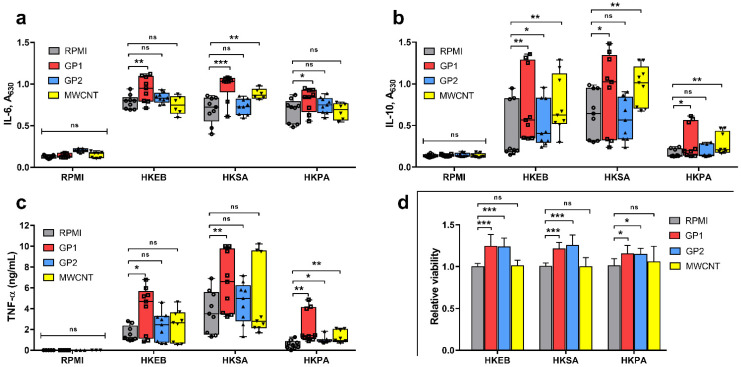
Cytokine response of primary monocytes to GPs (60 μg/mL) and MWCNTs (30 μg/mL) and heat-killed bacteria. (**a**) IL-6, (**b**) IL-10 and (**c**) TNF-α production. Cells were incubated in full RPMI and C-BNM for 24 h. After washing, cells were restimulated with HKEB, HKSA or HKPA for a further 24 h. Results were analysed separately using the paired Wilcoxon test, and data are presented as a boxplot from min to max with all points. *** *p* < 0.001, ** *p* < 0.01 and * *p* < 0.05, ns, not significant, highlights statistical significance as compared to corresponding control (RPMI). (**d**) Viability of monocytes after incubation with heat-killed bacteria. Data are normalised to control (RPMI) and reported as mean ± standard deviation. *** *p* < 0.001, * *p* < 0.05 highlights statistical significance as compared to corresponding control (RPMI).

The cytokine response of primary human monocytes was verified by the detection of IL-6, IL-10 and TNF-α production. None of the C-BNM alone induced a significant release of these cytokines after the initial 24-h exposition (Figure 4). These pre-treated monocytes, along with the control monocytes (incubated only in full RPMI), were further exposed to Gram-negative HKEB as a standard TLR4 and TLR2 agonist, HKPA as a TLR2 and TLR5 agonist and Gram-positive HKSA as an exclusive TLR2 agonist for another 24 h. As expected, treatment with heat-killed bacteria (10^7^ cells/mL) led to the production of IL-6, IL-10 and TNF-α. However, the pre-exposure of monocytes to GP1 resulted in the significantly increased secretion of these cytokines in response to all the used pathogens compared to control monocytes (Figure 4a–c). Pre-exposure to MWCNT led to the significantly increased release of IL-10 in response to all pathogens and increased production of IL-6 and TNF-α, predominantly in response to HKPA and HKSA, respectively. On the other hand, pre-exposure to GP2 slightly increased only TNF-α and IL-10 secretion, with no modulation of the IL-6 response to the studied pathogens compared to control monocytes.

#### 3.3.2. Viability

We also investigated the viability of treated monocytes using LDH assay. Treatment of control monocytes with heat-killed bacteria led to increased release of LDH (~10%), but monocytes that were pre-treated with C-BNMs preserved their viability without any additional release of LDH (Figure 4d). To avoid false-negative results, we performed an interference assay in which we compared lysates of cells previously treated with C-BNMs to untreated cells. No interference was detected.

### 3.4. Evaluation of Phagocytosis

**Figure 5:**
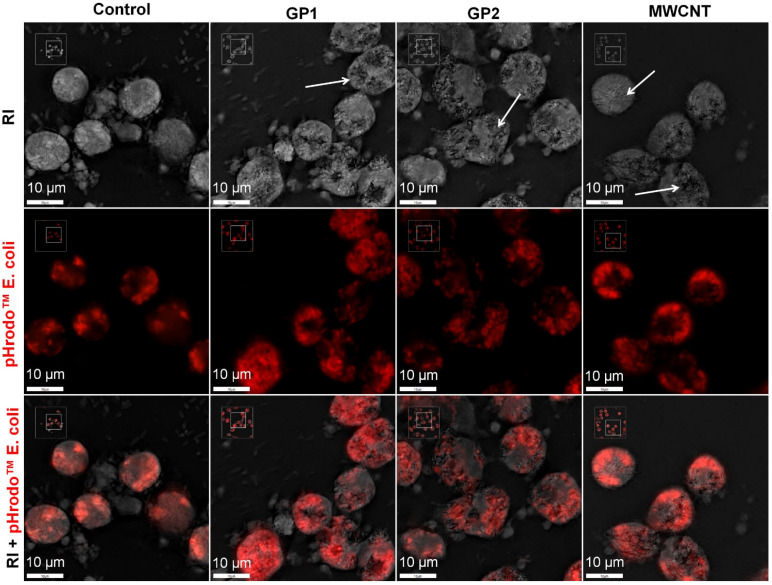
Internalization of EC (red) by primary monocytes pre-treated with GPs (60 μg/mL) and MWCNTs (30 μg/mL) (white arrows) compared to control after 3 h of exposure. Representative images taken using holotomographical microscopy. RI—refractive index.

For the evaluation of the intracellular C-BNMs’ effect on the phagocytosis of primary monocytes, we measured the phagocytosis activity using a pHrodo™ Red *E. coli* BioParticles™ (EC). Figure 5 shows micrographs of isolated monocytes with internalised C-BNMs and EC visualised by holotomographical microscopy with an epifluorescence module at wavelengths corresponding to the excitation spectrum of pHrodo^®^ dye. As the pHrodo^®^ dye fluorescence is observed only in acidic environments, only the EC present in phagosomes are visible. After 3 h of incubation with EC, we observed active phagocytosis in the untreated control and in cells with previously internalised GPs or MWCNTs. However, the cell observation showed slight changes between the control and monocytes, especially for both GPs. In this case, cells were better attached and showed enhanced incorporation of EC.

**Figure 6:**
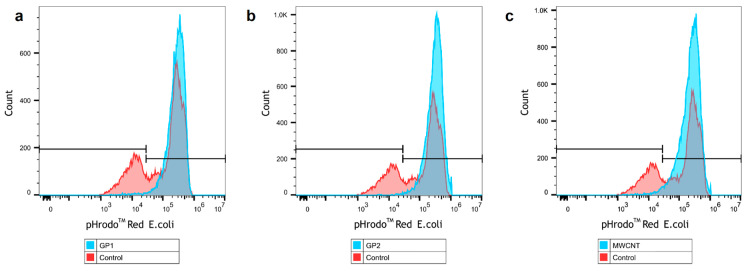
Representative flow cytometry images of phagocyted EC (200 μg/mL). Comparison of primary monocytes pre-treated by (**a**) GP1, (**b**) GP2, (**c**) MWCNTs (blue) and control without C-BNMs pre-treatment (red).

To confirm these findings, we analysed all cells by flow cytometry. Viability of measured cells was about 98% as verified using propidium iodide (data not shown). As shown in Figure 6, monocytes that previously engulfed C-BNMs phagocyted labelled EC particles at a higher percentage than the control. There was no difference between individual C-BNMs. The results are summarised in Table 2. To avoid false positivity, we analysed the autofluorescence of all C-BNMs. Neither of them interfered with the measurement (Figure S3).

### 3.5. Monocyte Differentiation to Macrophages

**Figure 7:**
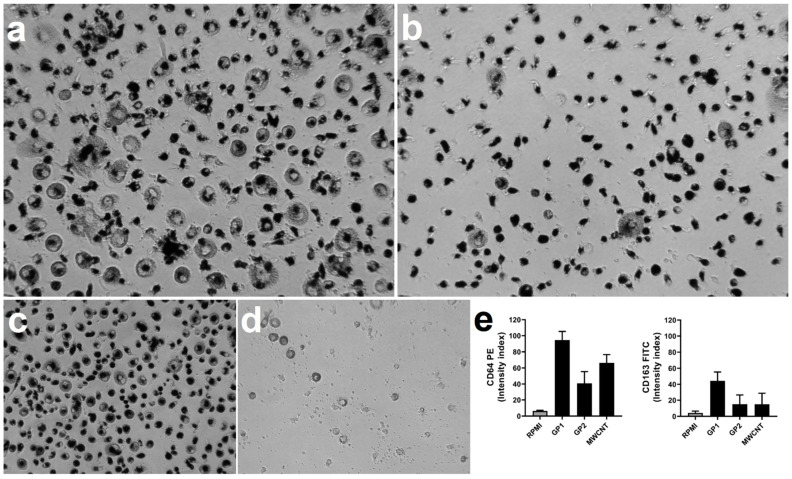
Morphology of cells after 24 h exposure and six days of differentiation with (**a**) GP1, (**b**) GP2, (**c**) MWCNTs and (**d**) RPMI. Magnification ×20. (**e**) Expression rate of CD64 and CD163 measured by flow cytometry. Data are normalized to control (RPMI) and presented as mean ± standard deviation. Indices were calculated according to unstained samples to eliminate autofluorescence.

To investigate whether the intracellular presence of different C-BNMs modulate spontaneous differentiation of monocytes to macrophages, after 24 h incubation with GPs (60 μg/mL), MWCNTs (30 μg/mL) or with full RPMI only, cells were washed and incubated with fresh medium for a further six days. On the seventh day, the cells were observed under an optical microscope. Monocytes cultivated in RPMI only (Figure 7d) ended up with a significantly reduced cell number compared with treated cells. These remained well attached to the surface of the well-plate for the entire duration of differentiation. Cells pre-treated with GP1 and MWCNTs (Figure 7a,c) were considerably larger, mostly with a round shape, while cells pre-treated with GP2 mostly remained smaller (Figure 7b).

To further investigate phenotype differences, we assessed the expression of CD64 and CD163 as markers of M1 and M2 macrophage polarization, respectively. To eliminate macrophage autofluorescence, unstained samples were measured as well (Figure S4). All monocyte differentiation resulted in positive expression of both markers; however, cells pre-treated with GP1 had the highest expression rate, followed by cells pre-treated with MWCNTs (Figure 7e). According to the results of co-expression of both CD64 and CD163, there was no clear distinction between macrophage polarisation.

## 4. Discussion

The induction of inflammation is considered to be one of the principal mechanisms of the cytotoxic effects of nanomaterials [31]. Unregulated inflammation leads to the disruption of homeostasis at the cellular and tissue levels. Therefore, evaluation of proinflammatory potential is an essential step in studies dealing with the safety and biocompatibility of nanomaterials. Many types of C-BNMs have been shown to have a capacity to induce acute and chronic inflammation [10,32,33,34,35]. Despite the large quantity of available data, there are still information gaps, notably in our knowledge of C-BNMs, which alone cause neither acute cytotoxicity nor the release of proinflammatory cytokines. An example of such nanomaterials are GPs [12,36,37,38,39]. In our previous study, tested GPs caused neither cytotoxicity nor the release of IL-1β through the activation of NLRP3 (Nod-like receptor family pyrin domain containing 3) in a THP1-null model. On the other hand, the co-exposure of cells to GPs and MDP led to a significantly stronger activation of NLRP3 in comparison to the control, which was treated with MDP only [11]. These results indicate that C-BNMs can modulate physiological cellular responses towards common antigen stimuli. Moreover, it leads to the conclusion that “harmless” nanomaterials may boost the stimulus of adsorbed PAMPs/DAMPs and therefore serve as a Trojan horse, a phenomenon underlining the necessity to confirm the sterility of nanomaterials [40]. To avoid misinterpretation of immunological data, all nanomaterials should be tested for contamination by molecules of biological origin, such as LPS having the character of PAMPs. It is a necessary step because immune cells, notably monocytes, are extremely sensitive to endotoxin presence due to their expression of high levels of TLRs [40,41].

Prior to our experiments, biological contamination had been evaluated using reporter cell-based assays, which are sensitive to TLR4 agonists (LPS) and TLR2 agonists, respectively (Figure 1d). The use of cell-based assays was preferred to the classical limulus amoebocyte lysate (LAL) assay as the LAL assay had previously been reported to often interfere with nanoparticles [42]. The inability of C-BNM to activate TLR4 and TLR2 also corresponded with the absence of proinflammatory responses after incubation with primary monocytes (Figure 4). Pristine GPs or graphene flakes appear to have insignificant immunogenic effects in general. Exposure to GPs initiated neither an inflammatory response nor oxidative stress in a 3D human lung model [12]. Studies on mouse bone-derived macrophages confirmed the absence of increased levels of TNF α, IL-6 and IL-1β [15]. The pulmonary administration of GP in rat and mouse models resulted in minimal inflammation, as well. On the other hand, these in vivo studies confirmed the presence of GPs in cells for a long time [13,37,39]. Despite the lack of direct immunogenic effects, their intracellular presence may modulate immune reaction towards different stimuli.

In this study, we have investigated the immunomodulatory effects on human primary monocytes. One of the main effector functions of monocytes is to promote inflammation. As previously stated, the leading mechanism of the proinflammatory effect of nanomaterials is NLRP3 activation and the subsequent release of IL-1β [11,22,32,34,43,44]. On the other hand, the release of IL-6, IL-10 and TNF-α, which is more typical of microbial stimulation, represents a physiological response towards common antigens. These cytokines play a vital role in the early stages of proinflammatory responses, cell signalling and mutual regulation; for this reason, the dysregulation of their release could lead to uncontrolled inflammation and damage of tissue [45]. The production of the abovementioned cytokines is mediated via cell membrane- and endosome membrane-associated (intracellular) TLRs. As evolutionary conserved structures, TLRs are supposed to recognize the products of various microbes. Therefore, cell stimulation by only one synthetic or purified ligand is likely to provide a limited answer. To obtain complex data, we used whole bacteria in the form of the Gram-negative HKEB (TLR4 and TLR2 agonist) and HKPA (TLR2 and TLR5 agonist) and the Gram-positive HKSA (TLR2 agonist), which mimic a more natural scenario. As expected, monocyte exposure to these bacteria (10^7^ cells/mL) led to proinflammatory responses characterised by the release of IL-6, IL-10 and TNF-α (Figure 4). The same concentrations of bacteria were used to test the immunomodulatory effects of C-BNMs.

Previous studies have confirmed that C-BNMs are quickly engulfed by macrophages and primary monocytes [11,15]. The main mechanism of C-BNMs’ entry into cells was found to be endocytosis, particularly phagocytosis [46]. Additionally, graphene microsheets were observed to enter cells through spontaneous membrane penetration [47]. The same mechanism was observed for nanodiamonds [32]. Subsequent intracellular distribution is dependent on the specific cell type. In the case of phagocytic cells, such as the activated THP-1 model, GP are usually present, enclosed in endosomes [11]. Observation of TEM pictographs suggests a similar situation for primary monocytes, especially when GP1 aggregates appear to be enclosed in large vacuoles (Figure 3a). Several studies also proved graphene occurrence in autophagosome-like vesicles [13,48,49]. Pristine CNTs are usually dispersed in cytoplasm without specific adherence to intracellular organelles. This corresponds with the finding of MWCNTs within monocytes (Figure 3c). Depending on shape and size, CNTs are often linked with the concept of frustrated phagocytosis [33,50]. Considering the shape of MWCNTs, their intracellular distribution eventually leads to nonspecific intracellular damage. Unlike GP, several studies have already confirmed lysosome disruption by CNTs, followed by cathepsin B release [11,22,35]. However, the viability of monocytes exposed to MWCNT was not affected, which in general rejects the hypothesis of cell homeostasis disruption (Figure 2c) [11].

Endocytosis and the subsequent distribution of MWCNTs and GPs did not cause any acute cytotoxicity and inflammation. However, it should be considered that phagocytosis is an active process that requires the complex reorganisation of intracellular compartments. Necessary reorganisation of cytoskeleton and subsequent signalling may induce the survival and differentiation of cells [51,52]. It is possible that the incorporation of nanoparticles serves as the initial signal leading to a signalling cascade that initiates differentiation even without the presence of an immunogenic stimulus or growth factors. Current studies have shown that monocytes previously exposed to non-cytotoxic concentrations of nanoparticles or pathogens undergo epigenetic changes, which partially depend on signalling in the mTOR pathway. This leads to switching into anaerobic glycolysis, which is related to the higher survival rate of cells [53,54]. It correlates with our results, where all tested C-BNMs promoted the survival of monocytes and their differentiation into macrophages (Figure 7). Another possible mechanism could be an induction of autophagy, which has been proven to have a significant role in monocyte differentiation and survival by blocking caspase 3-dependent apoptosis [55]. Autophagy has already been mentioned as a possible endpoint of graphene intracellular distribution [10,48,49]. Furthermore, this mechanism is also essential in M1/M2 polarisation [56,57]. On the other hand, autophagy usually results in supressing cytokine release, which we did not observe after the stimulation of pre-exposed monocytes by bacteria (Figure 4a–c).

Increased differentiation of monocytes pre-exposed to C-BNM could also explain their enhanced phagocytosis of EC, which we confirmed for all C-BNMs (Table 2). Differentiation is a highly complex process that requires molecular signalling, which influences the phagocytic activity of cells as well. In Figure 5, a slight difference in size between cells pre-treated by C-BNMs and control is already visible, which possibly indicates the early initiation of differentiation and subsequent enhanced phagocytosis (Table 2).

However, the increased levels of phagocytosis do not appear to be related to the production of higher levels of cytokines (Figure 4a–c). Although the intracellular presence of GP2 enhanced the phagocytic function of monocytes, there was only minimal modulation in proinflammatory response. Compared to that, monocytes pre-treated by GP1 produced significantly higher levels of all tested cytokines in response to all bacteria (Figure 4). The reason for modulation may lay in the intracellular stimulation of transcription factors, which are responsible for monocyte/macrophage polarization towards M1/M2 subtypes and partially correlates with the induction of autophagy [28,58]. Several studies also revealed that C-BNM could increase the proinflammatory response by a mechanism on the epigenetic level [29]. Lebre et al. found that pristine graphene flakes may program bone-marrow-derived macrophages in advance of an enhanced inflammatory response towards TLR agonists. They found that graphene flakes promoted the increased release of IL-6 and TNF-α by a mechanism called innate immune training [27]. The main concept includes a non-specific, augmented immune response to a secondary stimulus through metabolic and epigenetic changes of innate immune cells [59]. Compared to that, the prevailing release of anti-inflammatory cytokine IL-10 from monocytes pre-treated with MWCNTs suggests the induction of tolerance (Figure 4c). It may indicate an autoregulative mechanism since nanotubes alone may stimulate NLRP3 and IL-1β secretion [60,61]. Overstimulation or repetitive stimulation of primary monocytes could activate regulatory mechanisms and subsequently silence inflammation, thus preventing the disruption of homeostasis [62]. Additionally, it points to the preferred differentiation and polarization into M2 macrophages. However, we were not able to clearly distinguish the M2 polarisation (Figure 7e). Generally, C-BNMs have previously been shown to initiate M1/M2 polarization in a THP-1 macrophage model [28]. Laverny et al. also evaluated alterations in monocyte differentiation to dendritic cells by MWCNTs [26]. In any case, dissection of the M1/M2 phenotype based on CD markers is difficult as macrophages are highly plastic cells and the majority of CD markers co-exist in both populations [63]. As M1/M2 polarisation was not the main subject of this study, further experiments are needed to address this issue.

In conclusion, there are various mechanisms involved in monocyte modulation by GP1, GP2 and MWCNTs. The results of LDH assays, GSH/GSSG ratios (Figure 4) and PI staining (Table 2) imply that the modulation observed in C-BNM-stimulated monocytes was not mediated by cytotoxic effects. As all C-BNMs were prepared according to the same sonication protocol, the critical distinguishing factors appear to be the size and shape of the tested C-BNMs and their subsequent intracellular distribution. While GP1, with significantly smaller lateral dimensions (Figure 1a), formed aggregates inside the enormous endosomes (Figure 1a), GP2 was more scattered in the cytoplasm in small vesicles (Figure 2b). Evidently, the engulfment and distribution of GP1 would require huge cytoskeletal changes, which, as mentioned above, are strongly related to the modulation of a variety of signalling pathways, including changes in transcriptional levels [51,52]. Large graphene microsheets were found to disrupt cytoskeletal organisation in murine macrophages and in epithelial cells [47]. On the other hand, the effect could be indirect due to the simple oppression of the cytoplasmic content and the excess reorganisation of cytoskeletal fibres. It leads to the question whether the engulfment of GP influences the migration ability of cells. Studies focused on graphene oxide sheets showed the inhibition of the migration of A549 and HeLa cells by direct reaction with actin [64,65]. However, the possible disruption of cytoskeletal fibres by GPs seems to caused be their mechanical action [66].

## 5. Conclusions

The immunomodulatory effect of nanomaterials is a critical issue in the evaluation of nanomaterial safety. This is especially urgent for acutely non-cytotoxic nanomaterials and bio-resistant nanomaterials such as C-BNMs. Their persistence in organisms may, among other things, interfere with classical defensive immunological functions, leading to inappropriate immune responses towards common pathogens or disruption of their activation and polarisation. To address these effects, we have used MWCNTs and two types of GPs for pre-treatment of human primary blood monocytes, which were subsequently exposed to bacterial stimuli. These primary cells possess high sensitivity and differentiation potential, which makes them an ideal ex vivo/in vitro model for testing both responsiveness towards various stimuli and differentiation/polarisation changes. We confirmed that GP1 in particular, which alone caused no acute inflammation or cytotoxicity, increased monocyte reactivity towards bacterial stimuli. We also demonstrated that both GP and MWCNTs increased monocyte phagocytosis over a short time period and accelerated their differentiation towards macrophages. According to our data, cytoskeletal alteration caused by different incorporation of particles with a certain size and shape might be involved. Further studies will follow, including the differentiation of monocytes into their progeny, to expand our understanding of the mechanisms of interaction between C-BNMs and the immune system.

## Figures and Tables

**Table 1:** Physical properties of GPs and MWCNTs [11].

Nanomaterial	Particle Size (nm) (Z-Average)	PdI	Particle Size (nm) (Diameter)	Particle Size (nm) (Length)	Average *ζ*-Potential (mV) (in full RPMI)
GP1	178.5 ± 103	0.188			−8.52
GP2	332 ± 85	0.293			−10.8
MWCNT			110~200	≤10,000	−13.1

**Table 2:** Percentage of monocytes that phagocytosed EC after 3 h of exposure as measured by flow cytometry.

Pre-Treatment	Monocytes with EC (%)	Monocytes without EC (%)	Viability (%)
Control	62–72	27–38	96–97
GP1	85–99	2–18	98–99
GP2	82–98	1–15	98–99
MWCNT	82–98	3–18	95–98

## Data Availability

The data presented in this study are available upon request from the corresponding authors.

## Supplementary Materials

Supplementary Materials can be found at https://www.mdpi.com/article/10.3390/nano11102510/s1. Figure S1: Biological contamination—interference assessment; Figure S2: Representative gating strategy for the identification of primary monocytes in isolate; Figure S3: Flow cytometry images of autofluorescence of C-BNMs; Figure S4: Flow cytometry analysis of macrophage phenotype.
