# Supplementary material for: Carbon-Based Nanomaterials Increase Reactivity of Primary Monocytes towards Various Bacteria and Modulate Their Differentiation into Macrophages
Source: Nanomaterials (Basel). 2021 Sep 27;11(10):2510. doi: 10.3390/nano11102510 (PMC8537728; doi:10.3390/nano11102510)
Supplement: Supplementary file 1 [file nanomaterials-11-02510-s001.zip › nanomaterials-1371872-supplementary.pdf]

Supplementary Information

Carbon-Based Nanomaterials Enhance Reactivity of Primary Monocytes towards Various Bacteria and Modulate Their Differentiation into Macrophages

Tereza Svadlakova [1,2], Martina Kolackova[1], Radka Vankova [1], Rumeysa Karakale[1], Andrea Malkova[2], Pavel Kulich[3], Frantisek Hubatka[4], Pavlina Turanek-Knotigova[4], Irena Kratochvilova[5], Milan Raska[6], Jan Krejsek[1,*], and Jaroslav Turanek [1,4,5,6*]

1 Institute of Clinical Immunology and Allergology, University Hospital Hradec Kralove and Faculty of Medicine in Hradec Kralove, Charles University, 50005 Hradec Kralove, Czech Republic; svadlakovat@lfhk.cuni.cz (T.S.); kolackovam@lfhk.cuni.cz (M.K.); vankovr@lfhk.cuni.cz (R.V.); rumeysa.karakale@fnhk.cz (R.K.)

2 Institute of Preventive Medicine, Faculty of Medicine in Hradec Kralove, Charles University, 50003 Hradec Kralove, Czech Republic; Malka8AR@lfhk.cuni.cz (A.M.)

3 Veterinary Research Institute, 62100 Brno, Czech Republic; kulich@vri.cz (P.K.)

4 C2P NEXARS, 625 00 Brno, Czech Republic; frantisek.hubatka@nexars.com (F.H.), pavlina.turanek@nexars.com (P.T.)

5 Institute of Physics, Czech Academy of Sciences, 18200 Prague, Czech Republic; krat@fzu.cz (I.K.)

6 Department of Immunology, Faculty of Medicine and Dentistry, Palacky University Olomouc, 77515 Olomouc, Czech Republic; milan.raska@upol.cz (M.R.)

* Correspondence: jan.krejsek@fnhk.cz (J.K.); jaroslav.turanek@nexars.com (J.T.)

Biological contamination of C-BNM – evaluation of interferences

Experimental

HEK-Blue™-4 cells and HEK-Blue™-2 were separately seeded in flat bottom 96-well plates at the density of 5 x 10^4 cells per well and treated with non-cytotoxic levels of GP (60 μg/mL) and MWCNT (30 μg/mL) alone or in combination with ultrapure LPS (100 ng/mL, TLR4 agonist) or HKSA (10^7 cells/ mL, TLR2 agonist) for 24 h. LPS (100 ng/mL) and HKSA (10^7 cells/ mL) alone were used as controls. Absorbance was measured in a microplate spectrophotometer Synergy HTX (Biotek, Bad Friedrichshall, Germany) at 630 nm wavelength. Viability of cells exposed to C-BNMs was evaluated by LDH assay according to manufacturer's protocol.

Results

There was no cytotoxic effect of C-BNMs on HEK-Blue™-4 cells and HEK-Blue™-2 (Figure S1b). Also, none of the C-BNMs interfered with used cell-based assay, as reactivity towards control stimulants remain unchanged (Figure S1a).

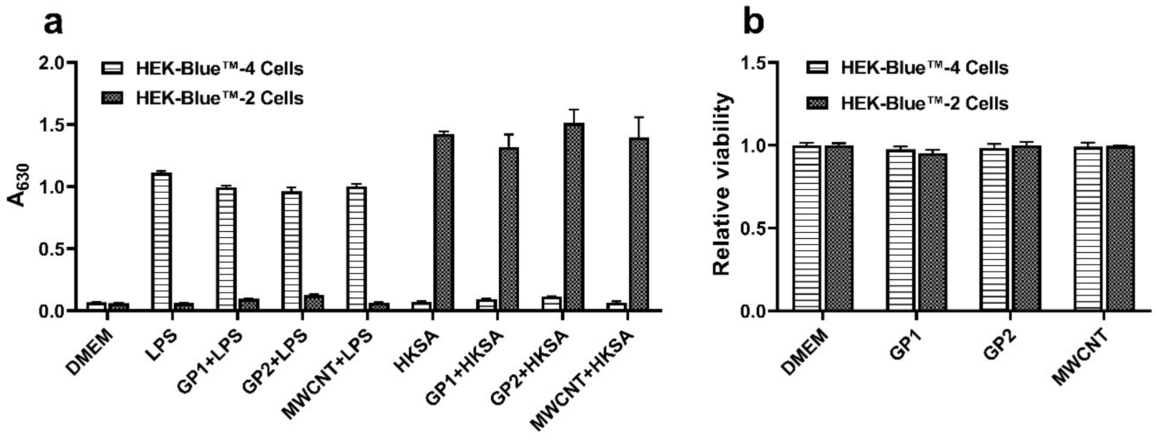

Figure S1. HEK-Blue™ exposure to C-BNM: (a) Comparison of HEK-Blue™-4 cells and HEK-Blue™-2 reactivity in response to cultivation medium (DMEM, negative control), C-BNM, LPS (TLR4 positive control), HKSA (TLR2 positive control) and C-BNMs spiked with LPS or HKSA. Data are presented as median with 95 % CI. (b) Viability of HEK-Blue™-4 cells and HEK-Blue™-2 after incubation with C-BNM. Data are normalized to control (DMEM) and reported as mean ± standard deviation.

Isolation of Monocytes

Experimental

Blood samples were collected into EDTA-treated BD Vacutainer® tubes (Becton Dickinson). Monocytes were isolated from these blood samples according to the manufacturer: following incubation with RosetteSep™ Monocyte Enrichment Cocktail, 50 μL/mL (STEMCELL Technologies Inc., Vancouver, Canada), gradient density centrifugation (1200g, 20 min, RT) was performed using Histopaque®-1077 (Sigma-Aldrich, St. Luis, MO, USA). The purity of isolated monocytes was evaluated by flow cytometry (Navios™, Beckman Coulter, Brea, KA, USA). Different subpopulations of leucocytes were distinguished based on expression of CD16 FITC (clone 3G8), CD56 PE (clone N901), CD19 ECD (clone J3-119), CD8 PC5.5 (clone B9.11), HLA-DR PC7 (clone Immu-357), CD3 APC (clone UCHT1), CD4 APC-Alexa 700 (clone 13B8.2), CD14 APC-Alexa 750 (clone RMO52), CD7 PB (clone 8H8.1), and CD45 KO (clone J33). All antibodies were purchased from Beckman Coulter (Brea, KA, USA). VersaComp Antibody Capture Beads (Beckman Coulter, Brea, KA, USA) were exploited for the correction of spectral overspill. Verification of optical alignment and optimization of the flow cytometer was done by Flow-Check Fluorospheres and Flow-Set Fluorospheres (Beckman Coulter, Brea, KA, USA). Kaluza (Beckman Coulter, Brea, KA, USA) software was used for sample analysis, including post-acquisition compensation. After isolation, cells were maintained in RPMI 1640 supplemented with 20% human autologous serum, 2 mM GlutaMAX and Primocin™. After 1 h of incubation, monocytes were carefully washed to get rid of the non-attached cells (~ 10% NK cells)

Results

The purity (~85%) of isolated monocytes was evaluated using Kaluza Analysis Software (Beckman Coulter, Brea, KA, USA).

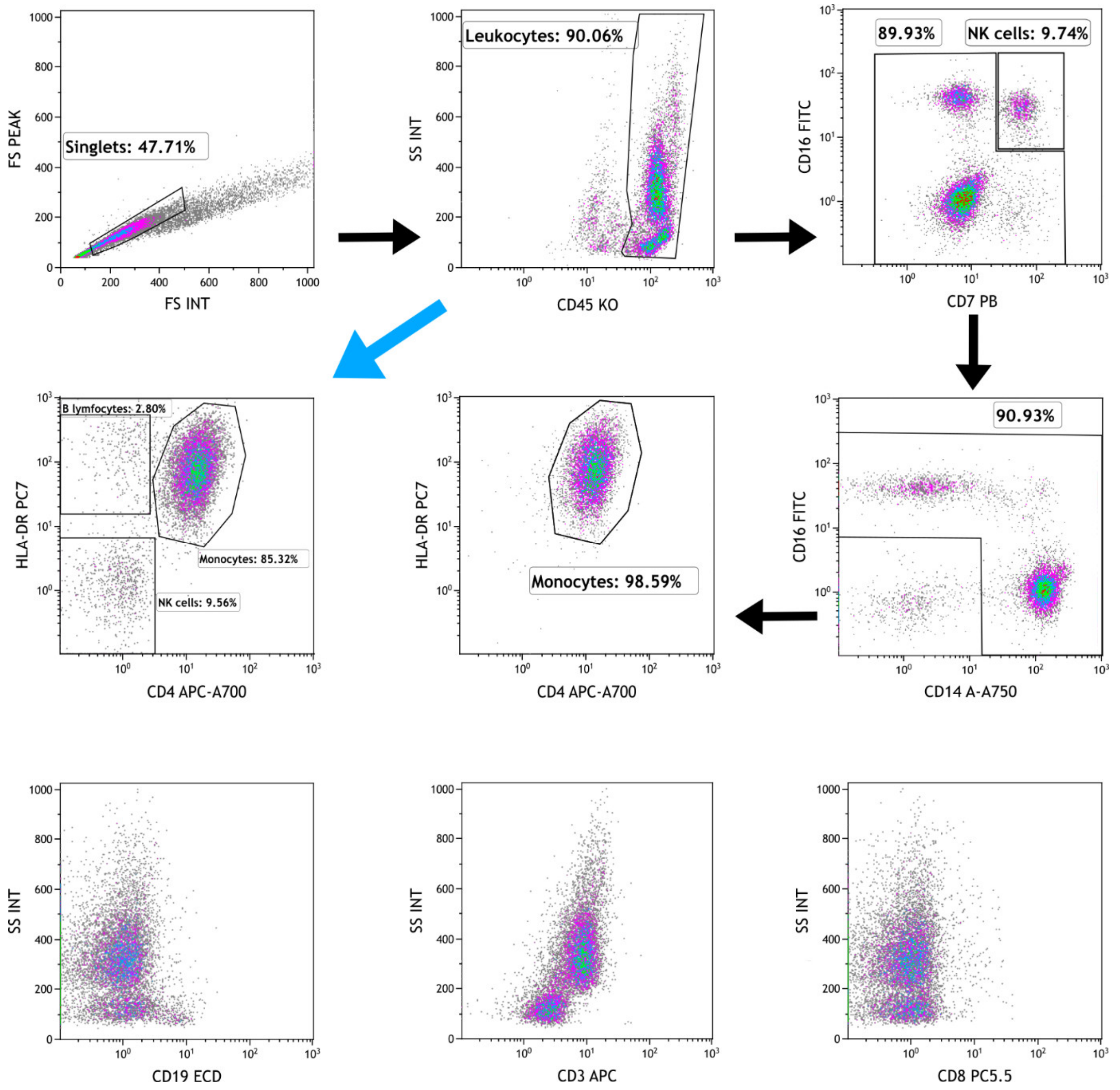

Figure S2. Representative gating strategy for the identification of primary monocytes in isolate; monocytes were identified among singlet cells; leukocytes were distinguished based on the side scatter characteristics and expression of CD45; natural killer (NK) cells (CD16, CD56 and CD7), T cells (CD3), and B cells (CD19, HLA-DR) we gated out; cells expressing CD14 and CD16 with co-expression of CD4 and HLA-DR were identified as monocytes. The purity ~85% of isolated monocytes was evaluated from the gate of leukocytes (blue arrow).

C-BNMs Autofluorescence Evaluation

Experimental

Monocytes exposed to C-BNM for 24 h were washed with PBS containing 1 mM EDTA, 1% BSA, 2% FBS and 0.1% NaN_3 (Sigma-Aldrich, St. Luis, MO, USA). Samples (30,000 events) were then acquired on Flow cytometer Navios™ (Beckman Coulter, Brea, KA, USA), using a 488 nm-argon ion laser, 402 nm-diode laser and 640 nm-HeNe laser to display autofluorescence in all channels.

Results

Figure S3 shows that there was no autofluorescence of C-BNMs treated cells compared to the control w/o C-BNMs

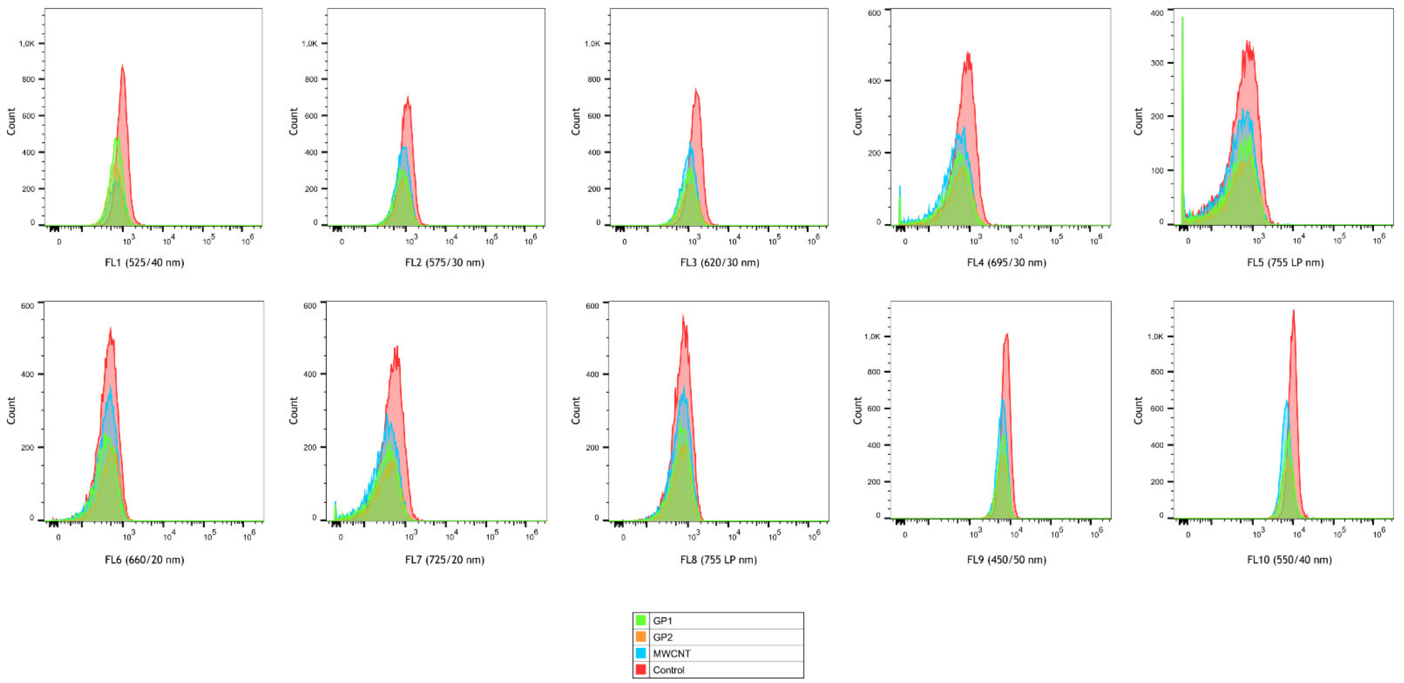

Figure S3. Flow cytometry histograms of autofluorescence of primary monocytes; untreated (red), with incorporated GP1 (green), GP2 (orange) and MWCNTs (blue).

Monocyte Differentiation to Macrophages-Phenotyping

Experimental

Detached cells were washed with PBS containing 1 mM EDTA, 1% bovine serum albumin (BSA), 2% FBS and 0.1% sodium azide (NaN_3) and stained with anti-CD64-PE (Beckman Coulter, Brea, KA, USA) and anti-CD163-FITC (Becton Dickinson, Prague, Czech Republic). Samples (60,000 events) were then analysed by Flow cytometer Navios™ (Beckman Coulter, Brea, KA, USA), using a 488 nm-argon ion laser and 525/40 FL1 and 575/30 FL2 channel.

Results

Due to various autofluorescence of differently treated samples, the expression (indexes) was calculated as a ratio of stained and unstained sample for each treatment individually, including the control without any treatment. Monocytes exposed to C-BNMs seem to differentiate faster and increase expression of CD64 and CD163 after 7 days of cultivation compared to control (Figure S4)

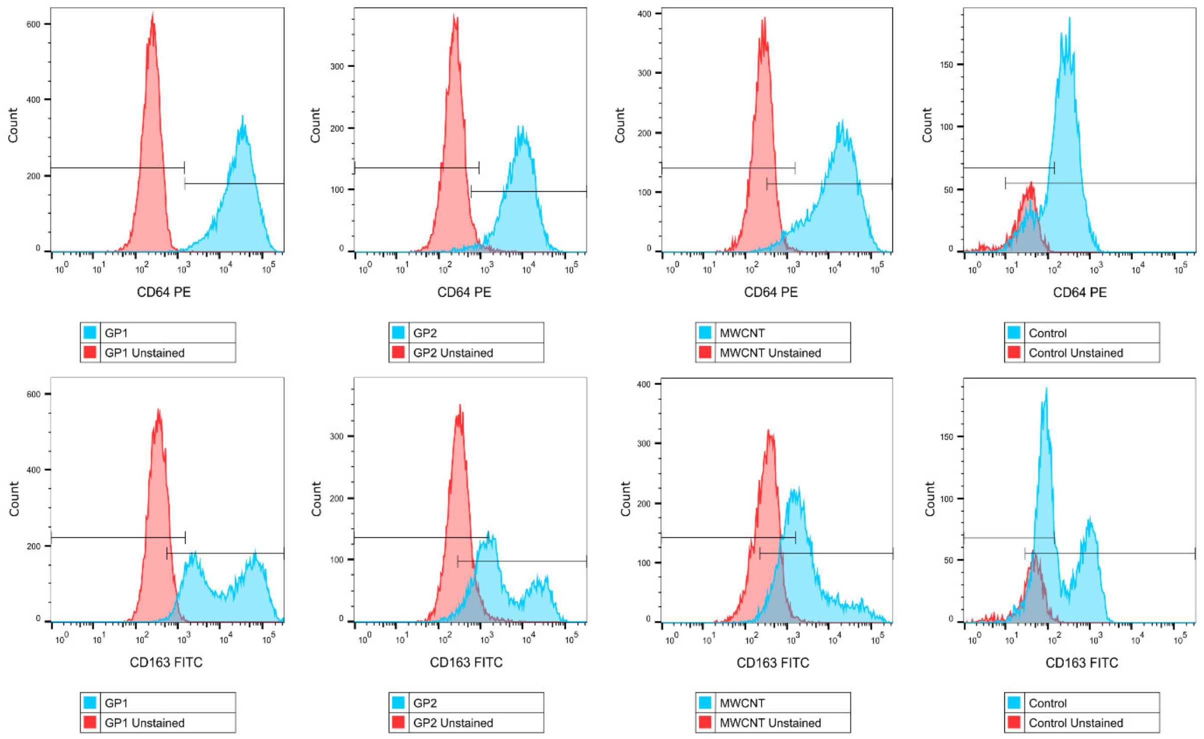

Figure S4. Flow cytometry histograms of expression of CD64 and CD163 in differentiating cells following the 7-day cultivation after their 24h incubation with C-BNMs or without incubation (control).
